# Food Insecurity in the Households of Polish Elderly: Diversity in the Perception of Its Causes by Demographic and Socioeconomic Characteristics

**DOI:** 10.3390/foods11203222

**Published:** 2022-10-15

**Authors:** Robert Gajda, Marzena Jeżewska-Zychowicz, Marzena Styczyńska, Małgorzata Agnieszka Jarossová

**Affiliations:** 1 Department of Human Nutrition, Faculty of Biotechnology and Food Sciences, Wrocław University of Environmental and Life Sciences, Chełmońskiego 37, 51-630 Wroclaw, Poland; 2 Department of Food and Consumption Market Research, Faculty of Human Nutrition, Warsaw University of Life Sciences, Nowoursynowska 166, 02-787 Warsaw, Poland; 3 Department of Marketing, Faculty of Commerce, University of Economics in Bratislava, Dolnozemská Cesta 1, 852 35 Bratislava, Slovakia

**Keywords:** food insecurity, elderly, causes of food insecurity, demographic characteristics, socioeconomic status

## Abstract

The aging of societies and the quality of life of the elderly may be accompanied by food insecurity. The aim of the study was to find the relationships between the perceptions of various causes of food insecurity, i.e., financial, social, health, and spatial, and then between those and the selected sociodemographic characteristics. The survey was conducted in late 2018 and early 2019 among 760 people aged 65 and older in two regions of Poland. Factor analysis with the use of principal component analysis (PCA) was used to identify the main causes of the prevalence of food insecurity. Cluster analysis using Ward’s hierarchical classification and logistic regression analysis were used to assess the relationship between the identified reasons for food insecurity, demographic characteristics, and socioeconomic status (SES). Two groups of causes that favor the experience of food insecurity among the elderly were identified, i.e., economic–social reasons and spatial–health reasons. They relate to such situations of food insecurity as concerns about food shortages, lack of staple foods, limited size or frequency of meals, and skipping meals. The high importance of economic–social (HE-S) reasons was associated with the low importance of spatial–health (LS-H) reasons, and conversely, the high importance of spatial–health (HS-H) causes was associated with the low importance of economic–social (LE-S) causes. HE-S and LS-H reasons were combined with low SES and residence in a city of more than 100,000 inhabitants. HS-H causes, on the other hand, were associated with LE-S causes and living in rural areas or towns of fewer than 100,000 inhabitants, as well as high SES. This specificity should be considered in the development of strategies and interventions aimed at reducing the phenomenon of experiencing food insecurity in the elderly population.

## 1. Introduction

Predictions of the growth of the elderly populations in different countries also apply to the Polish population [1]. In Poland, the share of the elderly (60 years and older) in the total population will increase from 25.9% in 2020 to 40.4% in 2050 [2]. Older people are exposed to various negative changes due to age, health, financial situation, etc. As a result, it can be expected that not only will the elderly population increase, but their quality of life may also decline due to the deterioration of general health and economic conditions [3,4], including, but not limited to, experienced food shortages or food insecurity [5]. In view of the economic and social costs of an aging population, there is a need to identify factors that increase the risk of age-related health problems and disability [6]. One such factor is food insecurity, understood as the limited or uncertain availability of nutritionally adequate and health-safe food or an uncertain ability to obtain acceptable food in a socially acceptable way [7].

Food insecurity refers to the economic and social problems of food shortages caused by various constraints, especially economic ones, and not by the adopted diet [8]. Even if food is economically available and accessible, other reasons can lead to food insecurity by reducing the ability to obtain it [9]. The main reasons for food insecurity in elderly households are related to poverty, social policy, and income [10]. Nevertheless, limited access to food among the elderly may be also related to living in a rural environment and to prevalent diseases [11,12,13].

Insufficient financial resources at the disposal of the elderly require the use of external assistance, thus social support, social relations, and social capital take on the responsibility [14,15]. Social support may reduce the risk of food insecurity, but this relationship is not entirely clear in light of the available knowledge [6]. Some previous studies have supported such a relationship [14,15,16], nevertheless some have also found no relationship [17] or even a negative relationship [18]. These contrasting results may be related to the magnitude of financial shortfalls and the various level of ability to use social assistance, especially in rural areas [19,20,21]. Moreover, some people who experienced financial problems benefited more from family assistance than from social assistance, which can be culturally determined [21,22]. Family assistance may also explain differences in perceptions of food security in the context of social assistance availability. In addition, perceived food insecurity may be determined by the distance from the place of residence to the place of sale [21,23,24,25] and limited mobility and transportation [16,26]. The limitations of the elderly in this regard can result from the lack of their own means of transportation or lack of skills to drive them, while the lack of public transportation exacerbates the problem [26]. The importance of the distance between the place of residence and the place of purchase also depends on the individual’s health, which may determine their mobility [27,28]. On the other hand, limited access to products such as fish and fish products, some fruits, beef meat, and some vegetables in the daily diet may contribute to an unhealthy diet and thus worsen health status [29,30,31]. Research by Morland et al., [32] suggested that living in areas with easy access to food sales’ points improves the quality of the diet. Moreover, access to supermarkets that offer food at lower prices allows more efficient use of financial resources at the disposal of the elderly. While the available research has focused attention on selected determinants of food security separately, knowledge of the interrelationships between these factors is still insufficient with regard to both objective and subjective indicators. The use of factor analysis allows for a more holistic view of the perception of food insecurity by those who are highly vulnerable to it due to their characteristics (e.g., older age, low socioeconomic status). The results will also enable a more comprehensive application in strategies to improve the quality of life of older people.

The negative consequence of food inaccessibility is the deterioration of the quality of life of the elderly [33,34,35,36]. In addition to such physical consequences as unhealthy eating and poor health, a person’s social and mental functioning deteriorates [37,38]. Therefore, research should take into account not only the actual extent of insecurity among the elderly but also subjective opinions about its occurrence and reasons, as they can affect the overall quality of life. The available data show that there is a real problem of food inaccessibility among the elderly [39], but also the problem of experiencing food insecurity is just as widespread. Food insecurity among the elderly is often connected to the lack of physical or health availability of food [40]. On the other hand, food safety in this group is determined not only by, among others, physical functioning and physical activity [27], socioeconomic status [37,41], social relations, social capital, and, to some extent, social support [14,15,42], but also by the distance of shops or supermarkets from the place of residence [23,24,25]. Therefore, it can be expected that similar reasons determine the perception of food insecurity, i.e., primarily financial restraints, but also factors related to social functioning, health, and physical accessibility to food. Based on the findings of previous research, it can be assumed that financial and social determinants are interrelated, as financial constraints can be reduced by social assistance, both public and from family members. On the other hand, health-related reasons such as poor health can exacerbate constraints related to the physical accessibility of food (long distance to a food outlet), hence increasing the importance of the latter in determining food insecurity. Thus, the aim of the study was to find the relationship between the perceptions of various causes of food insecurity, i.e., financial, social, health, and spatial causes, and then between those and the selected sociodemographic characteristics.

## 2. Materials and Methods

The research was carried out in two culturally and economically diverse regions in Poland. In 2019, the Świętokrzyskie region was the region with the lowest GDP (71.6% of medium GDP per capita), while the Śląskie region was characterized by high GDP (102.3% of medium GDP per capita) [43]. A cross-sectional survey was conducted from the beginning of October 2018 until the end of March 2019 among people aged 65 and older. The sample was selected using a non-probability sampling technique, i.e., snowball sampling. The choice of this method was based on the fact that it is cost-effective and helps to recruit reluctant individuals to the study. References from known individuals, rather than an unknown researcher, were helpful in recruiting the study group. A proprietary survey questionnaire was used for the study, with questions assessing food insecurity in elderly households adapted from the USDA (United States Department of Agriculture) recommended HFSS questionnaire (United States Household Food Security Survey Module) [44]. During the survey, 1150 questionnaires were distributed to 16 senior citizen clubs in the Świętokrzyskie and Śląskie regions. The recruitment criteria were age (65 and older) and consent to participate in the study. Those who gave their consent to participate in the study were asked to distribute the questionnaire to their acquaintances who met the age criterion. In total, 798 questionnaires were collected, of which 36 were eliminated due to non-responses. The final survey sample consisted of 760 people. The study was conducted in accordance with the Declaration of Helsinki [45]. Informed consent to participate in the study was obtained from all participants.

To assess the prevalence of food insecurity in elderly households, four questions about situations potentially conducive to feelings of food insecurity were asked. They referenced: (1) the concerns about food shortages in the household (*Have you been worried last month that your food will run out or has run out?*); (2) the shortage of staple food (*Have the staple food products,* e.g., *bread, butter, milk, eggs, etc., run out in your household last month*?); (3) the size or frequency of meals eaten (*Has the size or frequency of meals been lower in your household last month?);* (4) skipping meals (*Have you had to skip a meal last month*?). For each of these situations, four reasons for food insecurity were applied, i.e., (1) lack of financial resources; (2) living too far from the place where food is purchased (e.g., from a store, mall, bazaar, etc.); (3) lack of social support (e.g., from a social welfare center, food bank, from extended family, from friends, etc.); (4) health problems that make it difficult to purchase food (e.g., mobility problems, illnesses, disabilities, etc.). Responses were marked on a 6-point scale, with the following responses: 1, never; 2, once a month or less often; 3, several times a month; 4, once a week; 5, several times a week; 6, daily. Questions about the demographic characteristics of the study sample included gender, age, place, and region of residence.

The respondent’s socioeconomic status (SES) includes the following items: self-assessment of financial situation, use of family financial assistance, use of social financial assistance, and education. To learn about the self-assessment of the financial situation the two following questions were asked: (1) “*How do you assess your financial situation*?” with answers: below average (1 point); average (2 points); above average (3 points). (2) “*How do you assess the economic situation of your household?*” with the answers: I need to save to meet my basic needs (1 point); there is enough for my needs, but I need to save for larger purchases (2 points); there is enough without saving (3 points). One question was asked to assess family financial support: “*Do you get financial assistance from your family, including the family you live with?*” with the following answers: no, although I have financial problems (1 point); yes, because I have financial problems (2 points); no need, because my financial situation is satisfactory (3 points); yes, although I do not have financial problems (4 points). To determine social financial support, the question “*Do you use financial-related social assistance?*” was asked with the following answers: no, even though I have financial problems (1 point); yes, because I have financial problems (2 points); no need, because my financial situation is satisfactory (3 points); yes, even though I do not have financial problems (4 points). The response criteria to the question “What is your education?” was as follows: primary (1 point); vocational (2 points); secondary (3 points); higher education (4 points). The socioeconomic status (SES) of the participants was calculated according to the established procedure [46] separately for each participant by summing the scores obtained for each variable, i.e., self-assessment of financial situation, family and social support, and education. Cronbach’s alpha coefficient was used to assess the reliability of the input data for calculating the SES index [47]. Cronbach’s alpha coefficient was 0.781, thus confirming the accepted reliability of the measurement. Based on the tercile distribution of the SES index, groups of participants with low (lower tercile), average (middle tercile), and high SES (upper tercile) were distinguished.

Frequency distributions and cross-tabulations were used when categorical variables were analyzed. The chi-square test was used to examine the differences between categorical variables [48]. Factor analysis with the use of principal component analysis (PCA) was used to identify the main reasons for the prevalence of food insecurity. PCA used 16 variables that describe reasons for situations potentially conducive to feelings of food insecurity included in the study (four reasons for each of the four situations). Two components were extracted and rotated using the Varimax transformation. Components with an eigenvalue of 1 and a scree plot test were used to determine the number of components. Variables with factor loadings of at least 0.50 were taken into account. The factorability of the data was confirmed with the Kaiser–Meyer–Olkin (KMO) measure of sampling adequacy and Bartlett’s test of sphericity. The KMO value was found to be 0.892 and Bartlett’s test was significant at *p* < 0.0001.

For each factor (reason for food insecurity), two categories (decentiles) were distinguished, which were defined as the lower decentile (low, reason of low importance) and the upper decentile (high, reason of high importance). Cluster analysis using Ward’s hierarchical classification of variables and logistic regression analysis were used to assess the relationship between the identified reasons for food insecurity, demographic characteristics, and socioeconomic status (SES) [47]. Odds ratio (OR) values were calculated at the 95% confidence level. The reference group (OR = 1.00) was the lower decentile. A *p*-value of less than 0.05 was considered significant for all tests.

Statistical analysis was performed using STATISTICA statistical software (version 13.3 PL; StatSoft Inc., Tulsa, OK, USA; StatSoft, Krakow, Poland).

## 3. Results

### 3.1. Characteristics of Study Sample

The sociodemographic characteristics of the sample are displayed in Table 1. More than two-thirds of the participants in the study were women and those aged 65–74.

### 3.2. Relationship between Food Insecurity Factors and Demographic and Socioeconomic Characteristics

Table 2 shows the results of the principal component analysis along with the correlation coefficients between the causes of food insecurity, narrowed down to variables describing situations potentially conducive to feelings of food insecurity and each of the identified factors. Identified factors, i.e., the reasons for food insecurity were described as “economic–social” reasons (factor 1) and “spatial–health” reasons (factor 2). The “economic–social” reasons included concerns about food shortages, lack of staple foods, changes in the size and frequency of meal consumption, and skipping meals due to lack of funds or lack of financial social support. The “spatial–health” reasons were related to the same situations of food insecurity but caused by a long distance to food purchase locations or loss of health.

**Figure 1:**
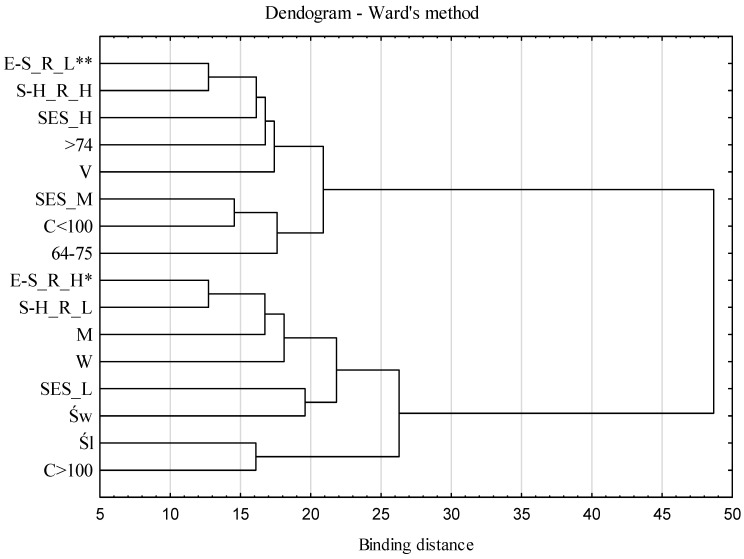
Dendogram showing the hierarchical classification of variables describing reasons for food insecurity, demographic characteristics, and socioeconomic status of respondents. * relationship between E-S_R_H—“economic and social” reasons of high importance, S-H_R_L—“spatial and health” reasons of low importance, W—women, M—men, Św—świętokrzyskie region, Śl—śląskie region, C > 100—a city with more than 100,000 inhabitants, SES_L—low socioeconomic status. ** relationship between S-H_R_H—“spatial and health” reasons of high importance, E-S_R_L—“economic and social” reasons of low importance, 65–74—age, >74—age over 74, V—village, C < 100—a city with less than 100,000 inhabitants, SES_M—average socioeconomic status, SES_H—high socioeconomic status.

The relationships between the identified factors (high/low intensity), demographic characteristics, and socioeconomic status (in tercile) are shown in Figure 1. Two clusters were distinguished with the use of Ward’s hierarchical classification of variables. The first cluster included respondents characterized by a high intensity of “economic–social” reasons, low intensity of “spatial–health” reasons, people living in large cities, and those with a low socioeconomic status. The group included both men and women and residents of both Śląskie and Świętokrzyskie regions. The second cluster included respondents declaring a high intensity of “spatial–health” reasons, a low intensity of “economic–social” reasons, residents of rural areas and small towns, and people of average and high socioeconomic status.

**Figure 2:**
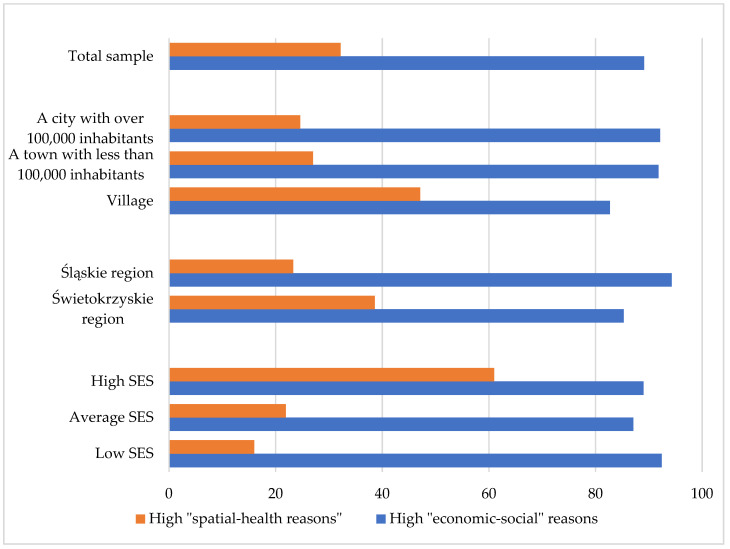
Importance of “economic–social” and “spatial–health” reasons for food insecurity according to selected demographic characteristics and socioeconomic status of the study sample (Chi-square test, *p* < 0.05).

The causes of food insecurity in selected demographic and socioeconomic groups are shown in Table S1 (as supplement). A high importance of “economic–social” reasons was declared by 89.1% of respondents, while a high importance of “spatial–health” reasons concerned 32.2% of respondents. Gender, age, and region of residence did not differentiate the importance of the two reasons causing food insecurity. In contrast, the largest number of people living in big cities indicated a high importance of “economic–social” reasons in causing food insecurity. More people living in rural areas, compared with those from cities, considered “spatial–health” reasons to be of high importance. Low socioeconomic status was associated with the high importance of “economic–social” reasons for food insecurity. In turn, high socioeconomic status was associated with the greater significance of “spatial–health” reasons for food insecurity (Figure 2).

The results of logistic regressions are presented in Table 3. They have demonstrated that people who declared a greater importance of “economic and social” reasons in determining food insecurity were more likely to live in cities with more than 100,000 inhabitants than in rural areas (OR, 1.89). In contrast, people who declared a great importance of “economic and social” reasons were less likely to display average SES (OR, 0.49) and high SES (OR, 0.57) compared with those with low SES. People who declared a great importance of “spatial–health” reasons in conditioning food insecurity were less likely to live in cities with more than 100,000 inhabitants (OR, 0.45) and in towns with less than 100,000 inhabitants (OR, 0.62) than in the countryside. Moreover, people who declared a high importance of these reasons were more likely to have average SES (OR, 2.03) and high SES (OR, 2.54) than low SES.

## 4. Discussion

The study addressed the causes of food insecurity among people aged 65 and older by looking at various situations indicative of food insecurity and various situations conducive to food insecurity, including financial, social, and health reasons, as well as the distance between where people live and where they buy food. The results of the study indicate that there are two groups of causes that favor the experience of food insecurity among the elderly, namely economic–social reasons (factor 1) and spatial–health reasons (factor 2). These reasons relate to the same situations of food insecurity in elderly households, i.e., concerns about food shortages, lack of staple foods, limited size or frequency of meals, and skipping meals. The importance of the identified group of reasons in determining food insecurity was differentiated by place of residence and socioeconomic status (SES), while there were no differences after accounting for gender, age, and region.

The identified group of situations conducive to experiencing food insecurity, i.e., economic–social and spatial–health reasons, are reflected in the results of previous studies [10,49,50]. Similar to previous findings, the results of the study indicated that economic–social reasons are crucial in developing food insecurity, and in the case of elderly people they are more important compared with spatial–health reasons. For about 80% of respondents, economic–social reasons were of great significance. The importance of economic factors, including income, but also financial resources for the level of food security in households has also been shown in many other studies [10,49,50,51,52]. In addition, Tarasuk et al., [49] indicated that insecure or inadequate access to food in households due to limited financial resources is on the increase, including in households in various developed countries.

Insufficient financial resources at the disposal of the elderly can make it difficult to meet even basic needs so that the need for outside assistance arises. Thus, social relationships, social capital, and social support gain importance [14,15]. However, the findings in this regard are so far inconsistent, which may be related to the size of financial shortfalls, the scope of social assistance, but also the willingness to use such assistance. It turns out that people experiencing food insecurity because of the lack of financial resources are both using and not using financial assistance, especially in the countryside [19,20,21]. Moreover, more people who had financial problems benefited from family support rather than from public social assistance, which can be culturally determined [21,22]. Preference for family support might also explain differences in feelings of food insecurity in the context of the availability of social assistance. The relationship between economic and social factors as determinants of food insecurity in the form of an identified factor seems justified, although the interrelationship requires further research to understand the mechanism of this interconnection.

“Spatial–health” reasons were important in determining food insecurity for about one-third of respondents. This is confirmed by the results of previous studies indicating that the distance from the place of residence to a food outlet not only determines the choice of a retail outlet [53,54] but also determines the ability of the elderly to purchase food [23,24,25]. The difficulties in purchasing food result from the distance to the supermarkets, which are typically located in the suburbs, and limited mobility and transportation [26]. In Poland, supermarkets are mostly located in cities, so their accessibility for people from rural areas is potentially more limited than for city residents, although this study did not assess participants’ mobility options. Nevertheless, lower accessibility to food products due to the commuting distance of the residence from the place where the food is purchased, especially among rural residents, was confirmed in another survey [55]. A large distance between the place of residence and the outlets resulted in a lack of products such as fish and fish products, some fruits, beef meat, and some vegetables in the daily diet [56]. Limiting the consumption of these products may lead to a worsening of the diet [30,56] and, as a result, to increasing the risk of various diseases [29,30,31]. The diseases of people who are 65 years old and older limit their physical fitness, which, combined with lack of transportation, loneliness, and distance to the grocery store, can significantly contribute to food insecurity [57,58].

The results obtained through the use of hierarchical classification indicate some differences in the reasons for experiencing food insecurity. The high importance of economic–social reasons was associated with limited spatial–health reasons and, conversely, the high importance of spatial–health reasons was associated with a limited significance of economic–social reasons. In addition, the high importance of economic–social reasons and the limited significance of spatial–health reasons were combined with low SES and residence in a city of more than 100,000 inhabitants. The greater importance of economic and social reasons in determining food insecurity in large cities may indicate that the cost of living exceeds the retirement and disability benefits received. Income shows a strong negative correlation with the lack of food security [59,60]. Since financial assistance protects against the decreasing capacity to procure food [61], activities focused on the improvement of the financial situation of older people living in cities are necessary. Physical accessibility to social welfare resources in urban settings appears to be greater (easier access to the office, greater IT skills), but limited funding for social welfare may be a key factor in addressing food insecurity. Another Polish study in a group of elderly people has shown that as many as one-fifth of the respondents did not use social assistance, despite their financial problems. Nonetheless, the region was more important than the place of residence in differentiating the use of such assistance, which may result from the different availability of such assistance, but also from the varying cultural backgrounds [55]. In this study, in particular low SES and living in a large city meant experiencing greater food insecurity, indicating the need to include this group in social assistance provided to the elderly. It is noteworthy that low SES and living in a rural environment did not prompt people to declare economic and social reasons for food insecurity, while other studies have linked living in rural areas to experiencing financial problems [19,20]. It seems that this may be determined by the specificity of the environment. On the one hand, family support, more frequent co-housing, and the availability of self-supplied food in rural settings can reduce food insecurity resulting from financial problems. However, the awareness of insufficient resources for effective functioning may cause disheartenment in people with financial problems [62] and also lower their expectations towards quality of life. In addition, the smaller extent of anonymity in the rural environment and reluctance to disclose one’s financial situation may also explain the lower importance of economic reasons for experiencing food insecurity.

The substantial significance of spatial–health reasons, on the other hand, was associated with a low intensity of economic–social reasons and residence in a rural area or in a city of fewer than 100,000 inhabitants, as well as high SES. The importance of spatial and health reasons in the rural environment should be associated with the distance to food outlets [37,63], limitations regarding the use of transportation facilities [28], and also with worse health [63,64,65]. Some previous studies have shown a stronger link between loss of health, especially mental health, and food insecurity than socioeconomic status and food insecurity [66], which could suggest that sick people, regardless of their socioeconomic status, may experience food insecurity. In this study, however, only high SES was associated with the increased importance of a spatial–health group of reasons for food insecurity. Food insecurity is not only strongly associated with poor health [13,67], but also with postponing needed medical care and medications [68,69]. Therefore, the spatial–health reasons that are more specific to the rural environment seem to be combined with the limited access to medical services, which leads to the abandonment or postponement of using medical services [67,70] and, as a result, to the deterioration of health. Worse health, with limited use of medical assistance, makes health reasons important and, together with spatial reasons, is an important cause of food insecurity in rural areas.

Gender, age, and region of residence did not differentiate the level of declared importance (high/low) of the two groups of reasons favoring food insecurity, nor increased the chance of declaring a high importance of both economic–social and spatial–health reasons. The results of previous studies indicate both the absence and the existence of differences in perceptions of food insecurity after accounting for certain sociodemographic characteristics. Darmon and Caillavet [71] and Leroux et al., [10] have found that gender and age do not differentiate older people in terms of food security, which also reflects obtained results. In contrast, a study by Tucher et al., [72] found that older people (aged 75–89) were less likely to experience food insecurity. Moreover, being female, having lower educational attainments, and being unmarried were all associated with higher rates of food insecurity [70].

The obtained results may find application in the development of strategies aimed at reducing food insecurity among the elderly; however, the study has some limitations. Firstly, the study was cross-sectional and thus the cause-and-effect relationship between the variables cannot be established. Moreover, this type of survey does not allow for noticing changes over time, hence it is difficult to assess to what extent the findings are derived from the state of the country’s economy. Secondly, due to the lack of representativeness of the study group (only two regions), the results of the study cannot be applied to the entire Polish population as well as to other populations due to a different cultural and economic context. Finally, the perception of food insecurity and the reasons for experiencing food insecurity were not considered in the context of the objective financial resources and/or the level of household poverty.

## 5. Conclusions

The study showed that experiencing food insecurity is linked to a number of causes, although some are interrelated. Health status and distance to food acquisition were one source of experiencing food insecurity, while lack of financial resources and lack of social support were the other source. Thus, the obtained results confirmed the link between social and financial causes, but also the relationship between health and spatial causes was revealed. These two identified groups of causes were inversely correlated with each other and they also differed in terms of the characteristics of older people experiencing food insecurity. Economic–social reasons for food insecurity were predominant in the study group and characterized respondents living in large cities and with low social status. Spatial–health reasons, on the other hand, were declared important in rural settings and smaller towns and among those with average and high SES.

Given that food insecurity is a complex phenomenon among older adults, future research should also include objective measures of food insecurity and its determinants in addition to subjective measurements to better identify the sources of food insecurity in the elderly population. Including both indicators in the survey at the same time would make it possible to explore the relationship between them and thus assess the usefulness of subjective opinions in evaluating the phenomenon. The identified factors as determinants of food insecurity among older people and the relationships between them seem reasonable, although understanding the mechanism of these links requires further research.

The obtained findings could be included in the development of strategies and interventions aimed at reducing the phenomenon of experiencing food insecurity. Public financial assistance is necessary for all elderly people who need it but is especially needed for low SES people living in metropolitan environments. On the other hand, the facilitation of physical accessibility to food, but also the facilitation of access to medical care, should be offered in the rural environment. It seems that the results of this study, despite a lack of representativeness, could also be applied to other sociocultural conditions, as can be judged by the lack of differences based on region.

## Figures and Tables

**Table 1:** Characteristics of the study sample.

Items	N = 760	%
Gender		
Women	527	69.3
Men	233	30.7
Age		
65–74 years	531	69.9
75 years and older	229	30.1
Place of residence		
Village	244	32.1
Town with less than 100,000 inhabitants	122	16.1
A city with over 100,000 inhabitants	394	51.8
Region		
Świętokrzyskie	443	58.3
Śląskie	317	41.7
SES index		
Low	225	29.6
Average	389	51.2
High	146	19.2

N—number of respondents.

**Table 2:** Factor load matrix for food insecurity reasons extracted using Varimax rotation.

Causes of Food Insecurity	Situations Potentially Conducive to Feelings of Food Insecurity	Identified Factors	
		Factor 1 “Economic–Social” Reasons	Factor 2 “Spatial–Health” Reasons
Lack of financial resources	Concerns about food shortages in the household	0.799 *	0.163
	Shortage of staple food	**0.787**	0.137
	Size or frequency of meals eaten	**0.784**	0.210
	Skipping meals	**0.760**	0.227
Living too far from the place where food is purchased	Concerns about food shortages in the household	0.317	**0.581**
	Shortage of staple food	0.320	**0.640**
	Size or frequency of meals eaten	0.437	**0.570**
	Skipping meals	0.497	**0.532**
Lack of social support	Concerns about food shortages in the household	**0.675**	0.378
	Shortage of staple food	**0.678**	0.353
	Size or frequency of meals eaten	**0.733**	0.337
	Skipping meals	**0.704**	0.356
Health problems that make it difficult to purchase food	Concerns about food shortages in the household	0.204	**0.800**
	Shortage of staple food	0.136	**0.822**
	Size or frequency of meals eaten	0.196	**0.842**
	Skipping meals	0.246	**0.729**
Variance Explained (%)		49.9	11.1
Total Variance Explained (%)		61.0	
Kaiser’s Measure of Sampling		0.892	

* factor loadings of at least 0.50.

**Table 3:** Odds ratios for food insecurity reasons of high importance according to selected demographic characteristics and socioeconomic status of the study sample.

Demographic Characteristics and Socioeconomic Status	Reasons for Food Insecurity of High Importance			
	“Economic–Social” Reasons *(Ref. ^a^ Low Importance)*		“Spatial–Health” Reasons *(Ref. Low Importance)*	
	OR ^b^	*p*	OR	*p*
Gender				
Men (ref. a women)	0.92 (0.58–1.47)	0.692	1.26 (0.79–2.01)	0.309
Women (ref. men)	1.09 (0.67–1.76)	0.692	0.80 (0.50–1.26)	0.309
Age				
75 years and older (ref. 65–74 years)	0.79 (0.49–1.27)	0.327	1.42 (0.91–2.20)	0.121
65–74 years (ref. 75 years and older)	1.29 (0.80–2.06)	0.317	0.74 (0.48–1.13)	0.150
Place of residence				
A town with less than 100,000 inhabitants (ref. village)	1.37 (1.89–2.10)	0.168	0.78 (0.49–1.23)	0.260
A city with over 100,000 inhabitants (ref. village)	**1.89** (1.15–3.03)	**0.012**	**0.45** (0.29–0.74)	**0.001**
A city with over 100,000 inhabitants (ref. town with less than 100,000 inhabitants)	1.36 (0.85–2.19)	0.196	**0.62** (0.38–0.99)	**0.049**
Village (ref. town with less than 100,000 inhabitants)	0.74 (0.49–1.15)	0.169	1.31 (0.83–2.08)	0.260
Village (ref. city with over 100,000 inhabitants)	**0.55** (0.34–0.89)	**0.012**	**2.23** (1.38–3.61)	**0.001**
A town with less than 100,000 inhabitants (ref. city with over 100,000 inhabitants)	0.72 (0.45–1.17)	0.196	**1.64** (1.00–2.69)	**0.049**
Region				
Śląskie (ref. świętokrzyskie)	1.19 (0.76–1.86)	0.485	0.76 (0.48–1.20)	0.217
Świętokrzyskie (ref. śląskie)	0.86 (0.55–1.35)	0.485	1.36 (0.85–2.16)	0.214
SES index				
Average (ref. low)	**0.49** (0.28–0.84)	**0.009**	**2.03** (1.27–3.26)	**0.004**
High (ref. low)	**0.57** (0.34–0.99)	**0.037**	**2.54** (1.61–4.02)	**<0.001**
High (ref. average)	1.29 (0.80–2.08)	0.318	1.28 (0.81–2.03)	0.309
Low (ref. average)	**2.11** (1.21–3.66)	**0.009**	**0.50** (0.31–0.80)	**0.004**
Low (ref. high)	**1.82** (1.07–3.12)	**0.029**	**0.40** (0.25–0.63)	**<0.001**
Average (ref. high)	0.80 (0.50–1.28)	0.328	0.79 (0.49–1.25)	0.309

^a^ reference group; ^b^ point estimate at 95% Wald confidence; *p*—significance level of the Wald’s test. Significant odds ratios are in bold.

## Data Availability

Data presented in this study are available on request from the corresponding author.

## Supplementary Materials

The following supporting information can be downloaded at: https://www.mdpi.com/article/10.3390/foods11203222/s1, Table S1. Importance of “economic-social” and “spatial-health” reasons for food insecurity according to selected demographic characteristics and socioeconomic status of the study sample.
