# Supplementary material for: Food Insecurity in the Households of Polish Elderly: Diversity in the Perception of Its Causes by Demographic and Socioeconomic Characteristics
Source: Foods. 2022 Oct 15;11(20):3222. doi: 10.3390/foods11203222 (PMC9601627; doi:10.3390/foods11203222)
Supplement: Supplementary file 1 [file foods-11-03222-s001.zip › foods-1908440-supplementary.pdf]

Table S1. Importance of "economic-social" and "spatial-health" reasons for food insecurity according to selected demographic characteristics and socioeconomic status of the study sample.

Demographic characteristics and socioeconomic status	Total sample		Reasons for food insecurity							
			„economic-social" reasons [a]				„spatial-health" reasons [b]			
			high		low		high		low	
	N	%	N	%	N	%	N	%	N	%
Total sample	760	100.0	677	89.1	83	10.9	245	32.2	515	67.8
Gender (*p≥0.05*)										
Women	527	69.3	470	89.2	57	10.8	164	31.1	363	68.9
Men	233	30.7	207	88.8	26	11.2	81	34.7	152	65.3
Age (*p≥0.05*)										
65-74 years	531	69.9	490	92.3	41	7.7	142	26.7	389	73.3
75 years and older	229	30.1	187	81.7	42	18.3	103	44.9	126	55.1
Place of residence (*p<0.05*)[a,b]										
Village	244	32.1	202	82.7	42	17.3	115	47.1	129	52.9
A town with less than 100,000 inhabitants	122	16.1	112	91.8	10	8.2	33	27.0	89	73.0
A city with over 100,000 inhabitants	394	51.8	363	92.1	31	7.9	97	24.6	297	75.4
Region (*p≥0.05*)										
Świetokrzyskie	443	58.3	378	85.3	65	14.7	171	38.6	272	61.4
Śląskie	317	41.7	299	94.3	18	5.7	74	23.3	243	76.7
SES index (*p<0.05*)[a,b]										
Low	225	29.6	208	92.4	17	7.6	36	16.0	189	84.0
Average	389	51.2	339	87.1	50	12.9	85	21.9	304	78.1
High	146	19.2	130	89.0	16	11.0	89	61.0	57	39.0

[a,b] Significant differences after accounting for demographic characteristics and socioeconomic status - Chi-square test, $p<0.05$.
